# Physical Characterization of Natural Straw Fibers as Aggregates for Construction Materials Applications

**DOI:** 10.3390/ma7043034

**Published:** 2014-04-11

**Authors:** Marwen Bouasker, Naima Belayachi, Dashnor Hoxha, Muzahim Al-Mukhtar

**Affiliations:** 1 Research Center in Divided Matter − CRMD, FRE CNRS 3520, 1b rue de la Férollerie, Orléans 45072, France; E-Mail: muzahim@cnrs-orleans.fr; 2 Multidisciplinary Laboratory for Research in Engineering Systems, Mechanical and Energy (PRISME), Polytech’Orléans, 8 rue Léonard de Vinci, Orléans Cedex 2, 45072, France; E-Mails: naima.belayachi@univ-orleans.fr (N.B.); dashnor.hoxha@univ-orleans.fr (D.H.)

**Keywords:** straw fibers, agricultural residues, microstructure, hygroscopic, sorption

## Abstract

The aim of this paper is to find out new alternative materials that respond to sustainable development criteria. For this purpose, an original utilization of straw for the design of lightweight aggregate concretes is proposed. Four types of straw were used: three wheat straws and a barley straw. In the present study, the morphology and the porosity of the different straw aggregates was studied by SEM in order to understand their effects on the capillary structure and the hygroscopic behavior. The physical properties such as sorption-desorption isotherms, water absorption coefficient, pH, electrical conductivity and thermo-gravimetric analysis were also studied. As a result, it has been found that this new vegetable material has a very low bulk density, a high water absorption capacity and an excellent hydric regulator. The introduction of the straw in the water tends to make the environment more basic; this observation can slow carbonation of the binder matrix in the presence of the straw.

## 1. Introduction

In Europe, buildings are responsible for more than 40% of the energy consumption and greenhouse gas emissions [1]. Thus, increasing building energy efficiency is crucial for the transformation of the UE energetic framework [2,3]. The article 9 of the European Directive establishes that, by 31 December 2020, all new constructions have to be nearly zero-energy buildings; for public buildings, the deadline is even sooner being at the end of 2018.

Since the national plan of action against climate change of February 2000 and the planning law and guidance for energy policy in July 2005, France is committed to fourfold its reduction of greenhouse gas emissions per citizen by 2050. This objective relates to almost a third of French real estate. The most effective way to insure these concepts is the use of eco-efficient thermal insulators. As a result, specialized engineers and researchers became attracted to the design and development of advanced materials in order to enhance and maximize energy performance and minimize energy consumption. Consequently, this will lead to a cost-effective, durable and eco-friendly construction material that will meet the global needs of thermal rehabilitation.

A good example is the natural fibers reinforced composites which are of interest as a replacement for conventional synthetic fibers reinforced polymers for some applications [4,5] in an increasing number of industrial sectors, especially automotive and aerospace [6]. These composites consist generally of two or more components with natural fibers in order to obtain specific characteristics such as high tensile, compressive strengths, and reduce shrinkage and cracking. In addition, they may be bio-degradable and completely or partially recyclable after their life cycle depending on the selected matrix [7].

Moreover, the natural fibers are derived from annually renewable resources [8], but it depends on countries and governments that are seeking non-oil reliant products [9]. Also, a recent surge of activity both in terms of industrial and fundamental research has been driven by environmental awareness of governments.

The advanced research of the last few years has shown that it is possible to produce high performance natural fiber composite based clay matrix [10], cement [11,12] and lime or polymer matrix capable of meeting any engineering demand in terms of strength and energy absorption capability [13–15]. Despite this growing interest, the applications of natural fibers are still limited to the non-structural field. Moreover, the disadvantages of natural fibers in some composites include the poor compatibility between fiber and matrix and the relatively high moisture sorption. However, for some construction or road applications where the concrete needs an ultra-ductile performance or better transport properties, the use of synthetic fibers have still been of interest in recent works [16–20]. For these applications, the good mechanical properties of natural fibers cannot compete with the high performance of synthetic fibers.

The biological degradation of the vegetable fibers is a major disadvantage of the use of these materials in the construction. The mould formation strongly depends on the water content of the natural fibers. High moisture content (above 20%) promotes the formation of mould [21]. Conversely, a straw whose water content is less than 15% by dry mass is assumed to be relatively stable and typically exhibits little evidence of microbial respiration [22]. Martinson [23] proposes to wrap the fiber and does not exceed water content of 15% in order to limit the development of mould of Orchardgrass hay.

No doubt, natural fibers, such as hemp, flax, cotton, jute, sisal, pineapple, cereal straw can be used in a variety of manners. However, there is a need for investigating the further properties of fibers.

Recent research studies are interested on the straw material in the form of bales. The straw bales actually used by the environment professionals proved to be an excellent construction material, as well as energy efficient and even fire-resistant. Some studies on straw bales have been carried for the effect of humidity, moisture content and thermal conductivity [24,25]. Unfortunately, the majority of studies on straw bales construction is at the scale of a straw bale, and need more accurate laboratory testing in order to understand the microscopic behavior of the straw fibers.

In this paper, a research study in the framework of a regional financed project entitled PROMETHE was done. One of the main objectives of this project is to propose a construction material based on a lime or cement matrix reinforced with cereal straw fibers for thermal rehabilitation.

The research program PROMETHE is a project partially sponsored by a network for the development of non-food biomass in the region “Centre” in France (named VALBIOM), which is the first participant to raise the issue of choice studied fibers before preparing the final straw based material. Wheat straw is likely to be the most widely used in construction because wheat production in the region “Centre” is more important; it reached 4.6 million tons to just 1.5 million for barley. Finally, the choice was to investigate the straw of wheat and barley of the same year (2010) and location, and two other straws of wheat for two different locations of 2011 in order to study the effect of the storage straw on its behavior. Therefore, in this study, four types of straw fiber are investigated: three wheat straws and a barely straw. The main objective is to study the morphological, physical and hydraulic properties of the four types of straw fiber.

After crushing particles, size distribution was determined by image analysis of scanned samples. The morphology of pores of isolated single fibers is examined by the electronic microscopy. The thermo gravimetric test is used to characterize the decomposition and thermal stability of materials. Finally, the retention water capacity, water absorption, and bulk density were measured.

## 2. Results and Discussion

### 2.1. Microstructure of the Straw Fibers

**Figure 1.**
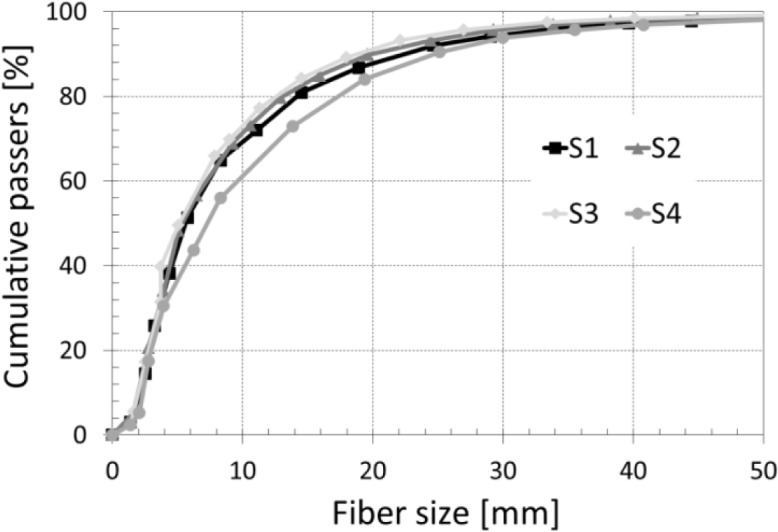
Fiber size analysis.

After the grinding, particle size analysis is carried out by image processing. The method allows obtaining the percentage for each fiber length. At the end of this operation, we drew the evolution of the cumulative passing *versus* the fiber length for the four straw types (Figure 1). Figure 1 shows that 95% of the straw fibers have a length of 30 mm. The maximum recorded length is 50 mm. It is noted that the three types of wheat straw (straws 1, 2 and 3) have the same distribution size. For barley straw, the particle size curve is flatter, indicating that the range (5–20 mm) is more important in ground barley straw.

**Figure 2.**
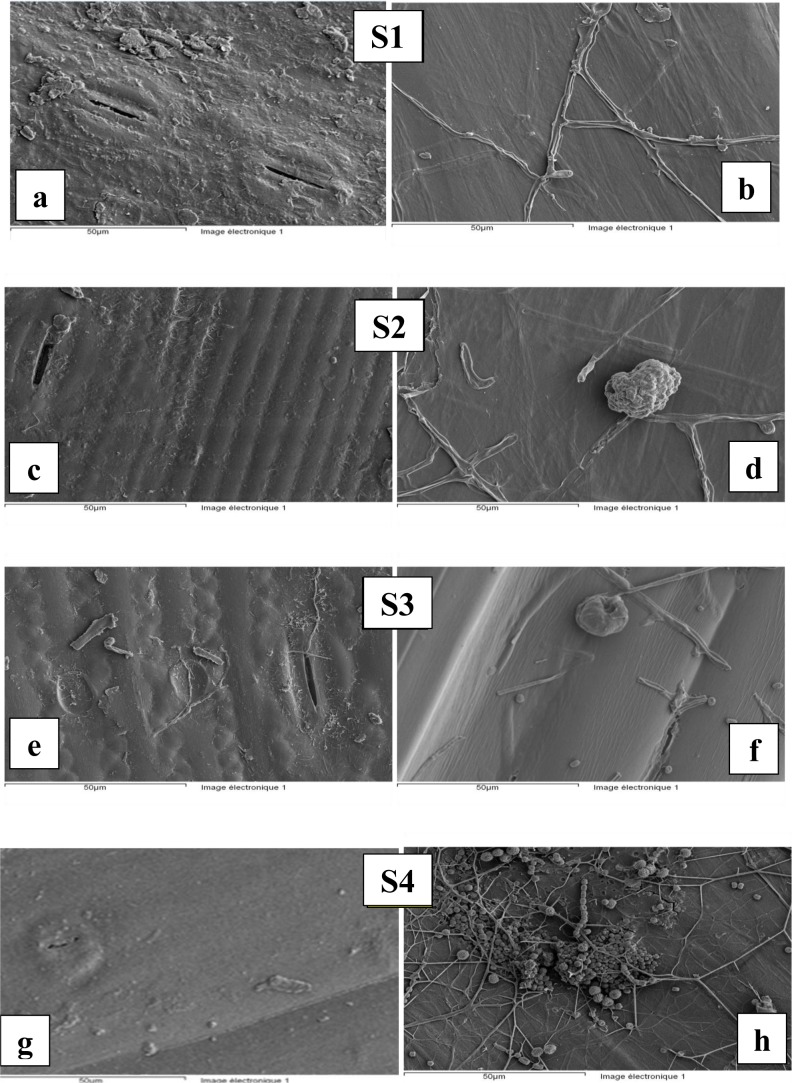
Outside surfaces (**a**) S1; (**c**) S2; (**e)** S3 and (**g**) S4 and inside surfaces (**b**) S1; (**d**):S2; (**f**) S3 and (**h**) S4 of the different straw fibers.

The outside and the inside surfaces (Figure 2) of the different straw particles have been studied by SEM. According to SEM photos, slits of 5–20 μm in length and a 0.5–2 μm opening are present on the outer surfaces of different straws. Barley straw denoted “S4” has the smaller slots, significantly lower than the three wheat straw fibers. The skin of the straw particles seems to be smoother. Unfortunately, this property has an adverse effect on the pull-out resistance of fibers. Indeed, roughness of the fiber is required to contribute to a better adhesion with the binder matrix (cement, lime, *etc.*) and consequently a better mechanical resistance. It is also noted that the skin of the different straws shows the presence of impurities coming from the storage environment.

The inside view (Figure 2) shows a very smooth surface with the presence of mould. SEM observation of the inner surface of the straw “S4” (Figure 2h) shows a very pronounced presence of mould. This finding may be due to storage conditions of barley straw which may be different from that of wheat straw.

Mould formation can cause harm to the fiber and the surrounding matrix. In order to avoid any damage, it is highly recommended to treat the fibers in order to eliminate moulds before insertion in the fiber reinforced composite. According to Bessadok, in order to eliminate mould, Alfa fibers are placed in a saline solution (30 g/L of NaCl) for 24 h and then rinsed and soaked for 24 h in an aqueous solution with 10% NaOH [26].

**Figure 3.**
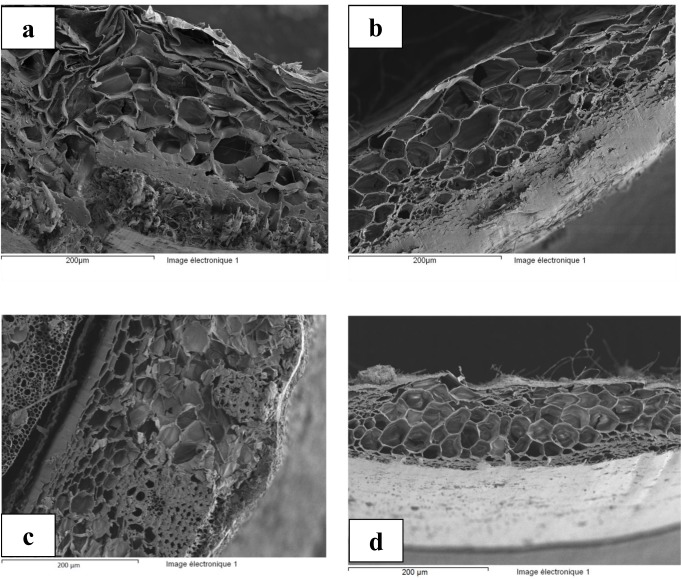
Cross sections of the different straw fibers (**a**) S1; (**b**) S2; (**c**) S3 and (**d**) S4.

Figure 3 shows the cross sections of the different straw fibers. These images show a complex structure with a very similar texture. From the outside to the inside, it includes sclerenchyma, parenchyma rings and vascular bundles included in the parenchyma.

The cross sections of the different straw fibers have a very dense exterior structure with variable thickness (between 90 and 130 μm) followed by a very porous structure. The porous structure includes hexagonal vessels. Their numbers decrease more and more towards the core of the straw particle. The diameter of these vessels varies from 7 to 20 μm. The microstructure showed a random pore size distribution of vascular bundle and an agglomerate of small cells on the outer surface.

### 2.2. Physical Properties

The physical properties namely bulk density, absolute density and void ratio are outlined in Table 1. Absolute density was measured by pycnometry using toluene as immersion liquid. Straw used for this test is completely dry. The Table 1 shows that the bulk density is much lower than many other lingo-cellulosic materials such as Hemp and wood chips (100 kg/m^3^). The absolute density is also much lower than some vegetable materials such as Hemp and wood aggregates. For instance, Hemp’s absolute density was nearly 1460 kg/m^3^ which represents 68% more than straw’s absolute density.

Table 1 shows that the absolute density of the four types of straw is nearly identical. In regards to the bulk density, barley straw (straw 4) has the highest value. It is 50% higher than that of wheat straw. The total porosity calculated from the absolute and the bulk densities is close to 96%. The high porosity justifies the interest to use this material in the manufacture of materials for thermal insulation.

### 2.3. Isothermal Sorption-Desorption Curves

**Figure 4.**
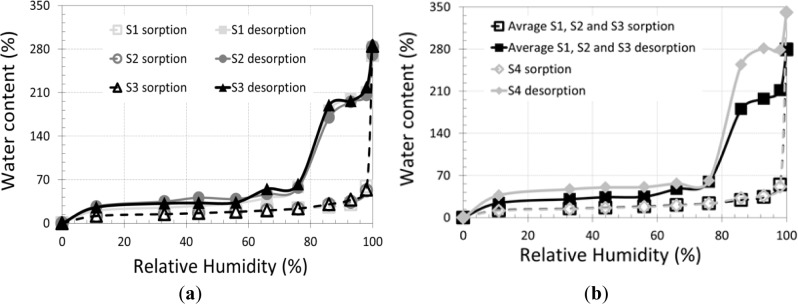
Sorption-desorption isotherms (**a**) 3 types of wheat straw; (**b**) comparison of wheat and barley straws.

Like most lingo-cellulosic materials, the equilibrium water content of plant fibers depends on the temperature, the physical properties and especially the relative humidity. The sorption-desorption isotherms which gave a relationship between the water content and relative humidity for the four types of straws are shown in Figure 4.

Figure 4a shows similar behavior of the different wheat straw fibers at saturation (sorption: curve in dashed lines). However, during desorption, different behaviors are highlighted. Behavior differs from one straw to another. Desorption kinetics of the barley straw “S4” (Figure 4b) is slower than the other three wheat straws, especially at relative humidity near saturation. There is also a difference between the sorption and desorption curves. This phenomenon is known as hysteresis. The hysteresis is to obtain for the same sample different water contents corresponding to the same relative humidity. The hysteresis of the “S4” straw curves is a little higher than the other straws, especially in low humidity. This can be explained by the outer surface of barley straw being denser (nanopores) and slightly porous compared to those of the three wheat straws (see Figure 3) which leads to a more significant loss of water from the vessels explained by classical Laplace capillary equation. This property gives the straw the role of an excellent hydric regulator that improves hygrothermal comfort.

### 2.4. Water Absorption Capacity

The water absorption coefficient is an important parameter in the mix design concrete based plant fibers. In fact, we will need to know the proportion of water absorbed by the straw to deduce the amount of water that reacts with the binder (cement or lime matrix). In this context, and in order to simulate the real conditions of mixing, the water absorption coefficient was studied as a function of time (during the first hour since the contact of straw–water) and as a function of the temperature (10, 20 and 40 °C).

**Figure 5.**
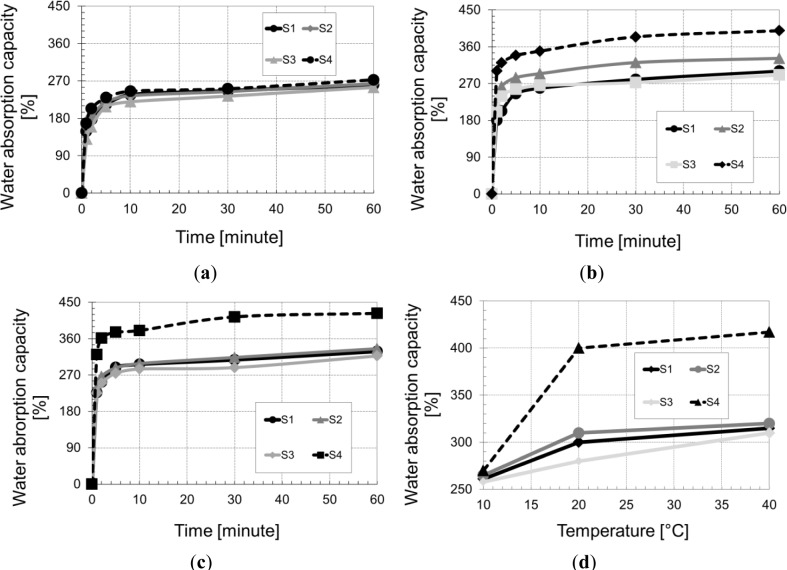
Water absorption capacity *vs*. time at different temperatures (**a**) at 10 °C; (**b**) at 20 °C; (**c**) at 40 °C; (**d**) water absorption capacity *vs*. temperature).

The general shape of the sorption curves (Figure 5a–c) of the straw is similar to other natural fibers such as hemp and flax. Curves describe the sorption process in two phases. The first phase corresponds to the Fick laws diffusion and the second phase is the non-Fickian diffusion which is controlled by the molecular relaxation process of the fiber:

- The first phase: the water absorption coefficient of the plant fibers is very high. The fibers are able to absorb a mass of water greater than their own weight. The curves of water absorption are linear in the initial phase when the intake of water results from the capillary action (due to the porous structure of the vegetable fibers). During this phase, which lasts 10 min, the water penetrates through the micro-pores capillary surface. Figure 5a–c show that during this period, the water content increases linearly up to about 70% of the total value obtained after 1 h of immersion.
- The second phase (non-Fickian diffusion phase): This deviation from Fickian diffusion is associated with change in the structure of the fibers under the influence of moisture, thus indicating the presence of internal stresses caused by the swelling of the material due to water absorption.

The studied straw fibers have a water absorption coefficient variable between 290% and 400% after 60 min immersion in water at 20 °C. These values are described as very high in comparison with those of flax and hemp. This feature is both harmful and beneficial. In the short term, a high absorption coefficient may create difficulties during the implementation of fresh material, due to the mobilization of a large part of the mixing water to the fibers. In the medium term, fibers saturated, which act as a water tank to pump the water to the binder matrix, reduce capillary depression and consequently reduce shrinkage.

According to Figure 5d, increasing temperature increases the water absorption of the studied fibers. This can be explained by an acceleration of the free water infiltration in the micro-pores. An increase of the temperature decreases the water viscosity and promotes the diffusion of the water in the fiber.

Increasing the temperature significantly increases the absorption coefficient of the straw 3 (barley) in relation to the other straw types (wheat). Between 10 and 20 °C, there was a sharp increase in the absorption coefficient. Beyond 20 °C, the absorption coefficient of the four straw types becomes quasi-stable.

### 2.5. PH and Electrical Conductivity

**Figure 6.**
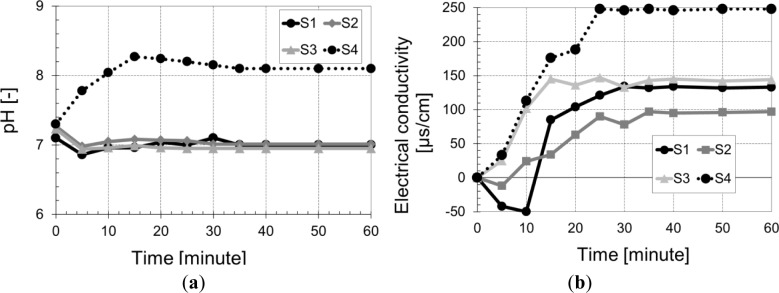
(**a**) pH and (**b**) electrical conductivity *vs*. time.

Figure 6 shows the evolution of the conductivity and pH of a solution of distilled water containing straw *versus* time. The conductivity curves (Figure 6b) have two shapes: a first accelerated phase up to 25 min, followed by a slowdown phase. This observation can be explained firstly by the dissolution of the cellulose initially present in the fibers, which passes into solution. Secondly, phenolic compounds initially localized in the plant cell walls would be released in the medium [27,28]. However, photo-oxidation of lignin, a majority polyphenol of plant walls, humic and fulvic acids could result in the synthesis of hydrogen peroxide (H_2_O_2_) in the solution [28–30] and thus increases the electrical conductivity. The particular type of lignin present in barley could explain the differences in electrical conductivity compared to the wheat straw. The conductivity of the straw named “S4” (barley) is 1.6 times greater than that of other wheat straw at 25 min. The evolution of pH *versus* time showed no significant change except for the straw “S4” (barley) where the pH increases to 8.3 after 15 min and then stabilizes. The increase in pH slowed carbonation and thereafter the setting time of the eco-friendly concrete.

### 2.6. Thermogravimetric-Analysis

**Figure 7.**
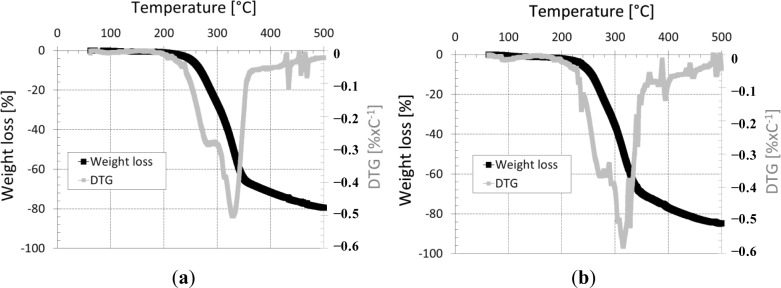
ATG and DTG analysis (**a**) wheat straw; (**b**) barley straw.

Figure 7a,b show the ATG and DTG curves of the two types of straws. From the superposition of results for the three types of wheat straw, in Figure 7a, only the results of the straw “S1” are presented. In a lingo-cellulosic material subjected to a temperature rise, generally hemicelluloses decompose first, followed by the decomposition of the cellulose and lignin. According to Figure 7a,b, we see that wheat and barley straws exhibit the same behavior in fire. Both figures show three major areas of weight loss on the TGA curves: 62–130 °C, 200–400 °C and >500 °C. The first peak observed is related to the dehydration of the straw fiber until 130 °C. Between 200 and 400 °C, two peaks on the DTG curves are observed. A first peak at 285 °C due to the decomposition of the fibers by depolymerization of hemicelluloses and pectins followed by a second peak at 329 °C, which reflects the degradation of the cellulose. Beyond 400 °C, low intensity peaks show the decomposition of lignin. The degradation temperature variation between barley and wheat straw is rather low.

## 3. Materials and Methods

### 3.1. Materials

This section describes the details of processing the fiber samples, preparation and the experimental procedures for their characterization. Four types of cereal straw were investigated: three types of wheat straw and one type of barley straw. Regarding wheat straw, they come from three fields located in the department “Eure et Loire”, a central region in France. The first two named “S1” and “S2” are from the harvest of 2011, while the third type “S3” is from the 2010 harvest. The fourth straw (barley straw) “S4” comes from the 2010 harvest. In the laboratory, dust is removed and then straws were ground into small particles by means of Bosch crusher plant (Bocsh AXT 23TC, Orleans, France). Ninety-eight percent of ground straw sizes are ranged between 1 and 35 mm. Finally, the straw particles were completely oven-dried at 60 °C for 72 h. All measurements were performed for three replicates samples and averaged to obtain final results.

### 3.2. Methods

In the absence of standards for measuring the most physical properties of plant fibers, all used methods will be detailed in the following:

#### 3.2.1. Straw Particle Analysis

Sieving analysis is not able to distinguish between length and width in the case of fibrous particle. Then, the image processing technique is designed to determine particle sizing.

A representative sample of 5 g of each straw type was randomly dispersed on a white paper of a surface equal to 2495 cm^2^. A high-resolution photo is then taken. The photo is then analyzed by an image processing program developed with Matlab software. The principle of the method is to scan the image line by line, recognize the fibers and then make a measurement depending on the length of the elements.

#### 3.2.2. Scanning Microscopy Analysis

Scanning electron microscope test (SEM) was performed in this study to observe the microstructure of various fibers. The high resolution scanning electron microscope equipment PHILIPS XL 40 ESEM (Philips France, Suresnes Cedex, France), was used for microscopic observation. After 72 h of drying, the sample is directly observed without any treatment. In this study, we focus particularly on the longitudinal and transversal surfaces of each fiber.

#### 3.2.3. Water Absorption Capacity

The water absorption capacity of straw fibers and the absorption kinetics were measured on a straw sample of 50 g completely dried (at 60 °C for 3 days). The water absorption coefficient was determined by submerging the samples in the water at several times (1, 2, 5, 10, 30 and 60 min). In order to study the effect of temperature on the kinetics of water absorption, three temperatures (10, 20 and 40 °C) were used. After wetting, the fibers were superficially dried using absorbent paper to remove the inter-fiber water and the water absorbed on their surface. The samples are weighted before and after wetting. The absorption coefficient in percentage was calculated using the following relationship:

$$WA(\%)=\frac{m_{1}-m_{2}}{m_{2}}\times 100\%$$ (1)

where *m*_1_ the weight of is wet fiber and *m*_2_ is the weight of dry fiber.

#### 3.2.4. Water Characteristic Curve (Sorption-Desorption Isotherms)

This test is inspired from the standard NF EN 12571 [31] for porous materials used in construction. A fundamental property of building materials is its ability to attract water at various water contents and suctions. To obtain the water characteristic curve, samples were placed in desiccators at various suctions, using different relative humidity values (from 11% to 98%). Relative humidity was fixed via a chemical solution with a known saline solution concentration. For each measurement, which means inside each desiccator, five plastic capsules containing straw samples were put inside each desiccator. Straw samples’ weight was measured using a digital balance with 0.001 g as accuracy. The equilibrium was reached if difference in straw mass is less than 0.05%. During the tests, the temperature is maintained constant (20 °C). The straw behavior *versus* the relative humidity was studied in the case of the sorption (the starting point is a dry sample) and in the case of desorption (the sample is initially saturated).

#### 3.2.5. PH and Electrical Conductivity

The pH of the solution is measured by a pH meter type “Consort C831” (Consort bvba, Turnhout, Belgium). The pH meter is firstly calibrated using pH solutions of 0, 7 and 14. PH was evaluated by dipping 5 g of a finely ground straw sample in a beaker of 200 mL capacity. Then, 100 mL of distilled water is added in order to respect a mass ratio of liquid straw equal to 20. After shaking, the straw is kept immersed in distilled water for 15 min. Every 5 min, a measure is taken until the stabilization (the difference in pH value is less than 0.05%).

With respect to conductivity, an electrode is connected to a conductivity meter type “Consort C3010”. The electrode is calibrated using a KCl solution of 0.1 mol/l (λ = 12.88 mS/cm). This unit can monitor and record changes in the conductivity of the straw suspension over time.

#### 3.2.6. Thermo-Gravimetric Analysis

Thermal decomposition was observed in terms of global mass loss by using a SE-TARAM ATG-DSC 111 thermo gravimetric analyzer (SETARAM Instrumentation, Caluire, France). This apparatus detects the mass loss with a resolution of 0.1 mg as a function of temperature. The samples were evenly and loosely distributed in an open sample pan with an initial sample amount of 5 mg. The temperature change was controlled from room temperature (22–500 °C) at a heating rate of 10 °C/min. Three specimens were used to check repeatability.

## 4. Conclusions

The results analysis of this study on the microstructural and physical characterization of four types of straw fiber for the purpose of eventual use in building insulation materials highlighted the following key points:

– There were significant differences between varieties (barley and wheat), whereas the differences between year of harvest were small.
– The sorption-desorption isotherms shows that the straw is an excellent hydric regulator that improves hygrothermal comfort of the building.
– The straw present a high water absorption coefficient compared with that of other vegetable fibers. At 20 °C, straw absorbs an average weight of water three times larger than its own weight. This value reaches four times for barley straw. This fact makes straw more sensitive *versus* water.
– The introduction of the straw in the water tends to make the environment more basic, this observation can slow carbonation of the binder matrix in the presence of the straw.
– The skin of the straw particles seems to be smooth. Unfortunately, this property has an adverse effect on the mechanical strength of final materials based straw fiber.

## Figures and Tables

**Table 1.** Densities and porosities of the different types of fibers.

Straw No.	Bulk density (kg/m^3^)	Absolute density (kg/m^3^)	Porosity (%)
Straw 1	30	871	96
Straw 2	33	867	96
Straw 3	25	865	97
Straw 4	47	870	94
